# Supplementary material for: The interactome of intact mitochondria by cross-linking mass spectrometry provides evidence for coexisting respiratory supercomplexes
Source: Mol Cell Proteomics. 2017 Dec 8;17(2):216–32. doi: 10.1074/mcp.RA117.000470 (PMC5795388; doi:10.1074/mcp.RA117.000470)
Supplement: Supplemental Data [file supp_RA117.000470_133922_0_supp_23978_fzffwf.zip › spectra_annotation/mito_spectra_annotation/18-1-6-1-6-1.pdf]

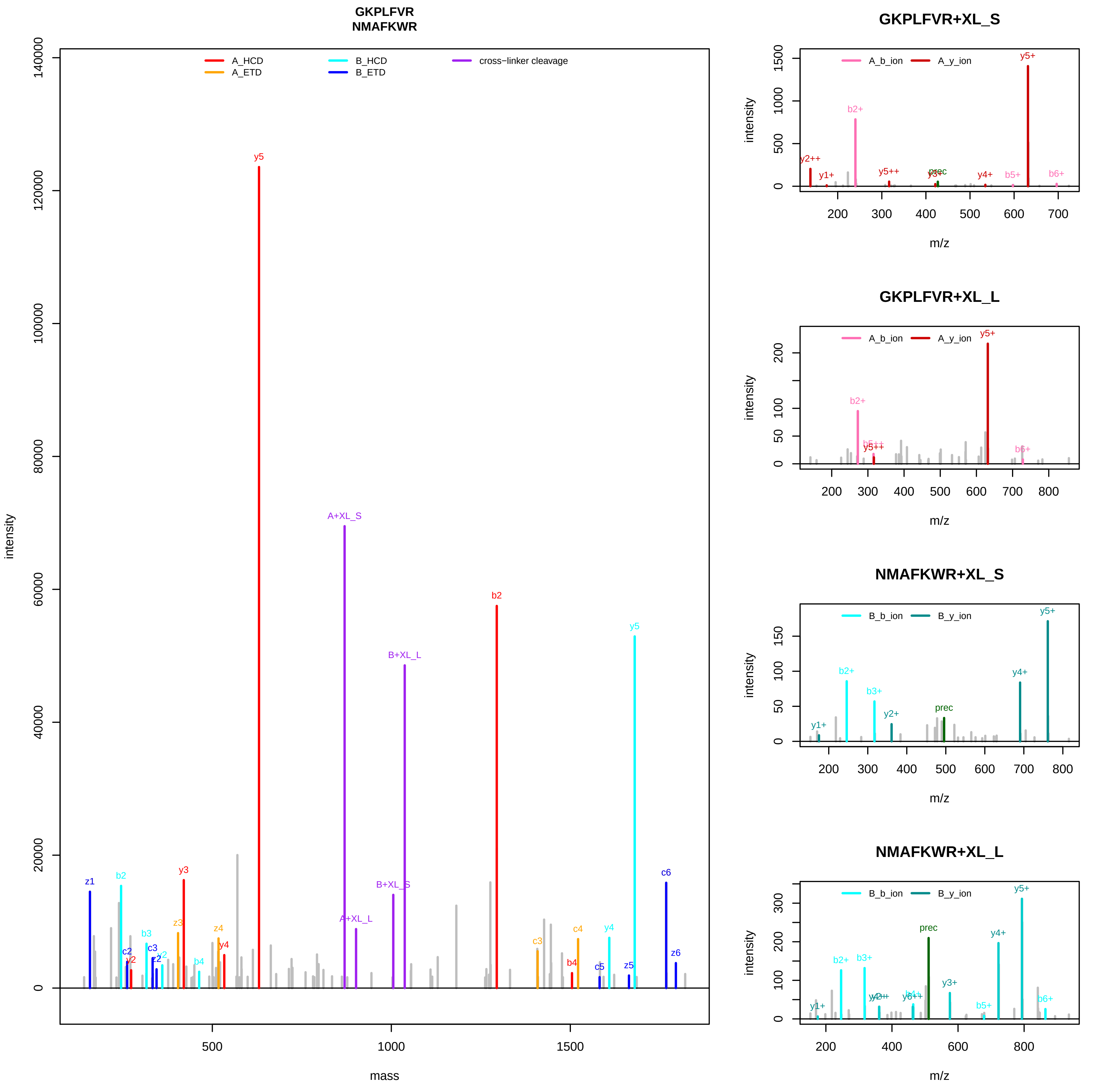

GKPLFVR
NMAFKWR
A_HCD
A_ETD
B_HCD
B_ETD
cross−linker cleavage
intensity
mass
y5
A+XL_S
B+XL_L
b2
y5
A+XL_L
B+XL_S
z1
b2
c2
y2
b3
c3
z2
y2
z3
y3
b4
z4
y4
c3
b4
c4
c5
y4
z5
c6
z6
0
20000
40000
60000
80000
100000
120000
140000
500
1000
1500
GKPLFVR+XL_S
A_b_ion
A_y_ion
y2++
y1+
b2+
y5++
prec
y3+
y4+
b5+
y5+
b6+
m/z
GKPLFVR+XL_L
b2+
b5++
y5++
y5+
b6+
NMAFKWR+XL_S
B_b_ion
B_y_ion
y1+
b2+
b3+
y2+
prec
y4+
y5+
NMAFKWR+XL_L
y1+
b2+
b3+
y2++
y6++
b4+
prec
y3+
b5+
y4+
y5+
b6+
